# Supplementary material for: Novel Fluorescence-Based Methods to Determine Infarct and Scar Size in Murine Models of Reperfused Myocardial Infarction
Source: Cells. 2024 Sep 30;13(19):1633. doi: 10.3390/cells13191633 (PMC11475149; doi:10.3390/cells13191633)
Supplement: Supplementary file 1 [file cells-13-01633-s001.zip › cells-3192317-SI.pdf]

Supplementary Material

Novel fluorescence-based methods to determine infarct and scar size in murine models of reperfused myocardial infarction

Ashley Duplessis [1,†], Christin Elster [1,†], Stefanie Becher[1], Christina Engel[1], Alexander Lang[1], Madlen Kaldirim[1], Christian Jung[1,2], Maria Grandoch[2,3], Malte Kelm[1,2], Susanne Pfeiler[1] and Norbert Gerdes[1,2,*]

[1] Division of Cardiology, Pulmonology, and Vascular Medicine, Medical Faculty and University Hospital, Heinrich Heine University, Düsseldorf, Germany; ashley-jane.duplessis@uni-duesseldorf.de (A.D.); christin.elster@hhu.de (C.E.); stefanie.becher@med.uni-duesseldorf.de (S.B.); christina.engel@hhu.de (C.E.); lang@hhu.de (A.L.); madlen.kaldirim@med.uni-duesseldorf.de (M.K.); christian.jung@med.uni-duesseldorf.de (C.J.); malte.kelm@med.uni-duesseldorf.de (M.K.); pfeiler@hhu.de (S.P.)

[2] Cardiovascular Research Institute Düsseldorf, (CARID), Medical Faculty, Heinrich Heine University, Düsseldorf, Germany; maria.grandoch@uni-duesseldorf.de

[3] Institute of Translational Pharmacology, Medical Faculty, Heinrich Heine University, Düsseldorf, Germany

* Correspondence: gerdes@hhu.de; Tel.: +49-211-81-05028

† These authors contributed equally to this study.

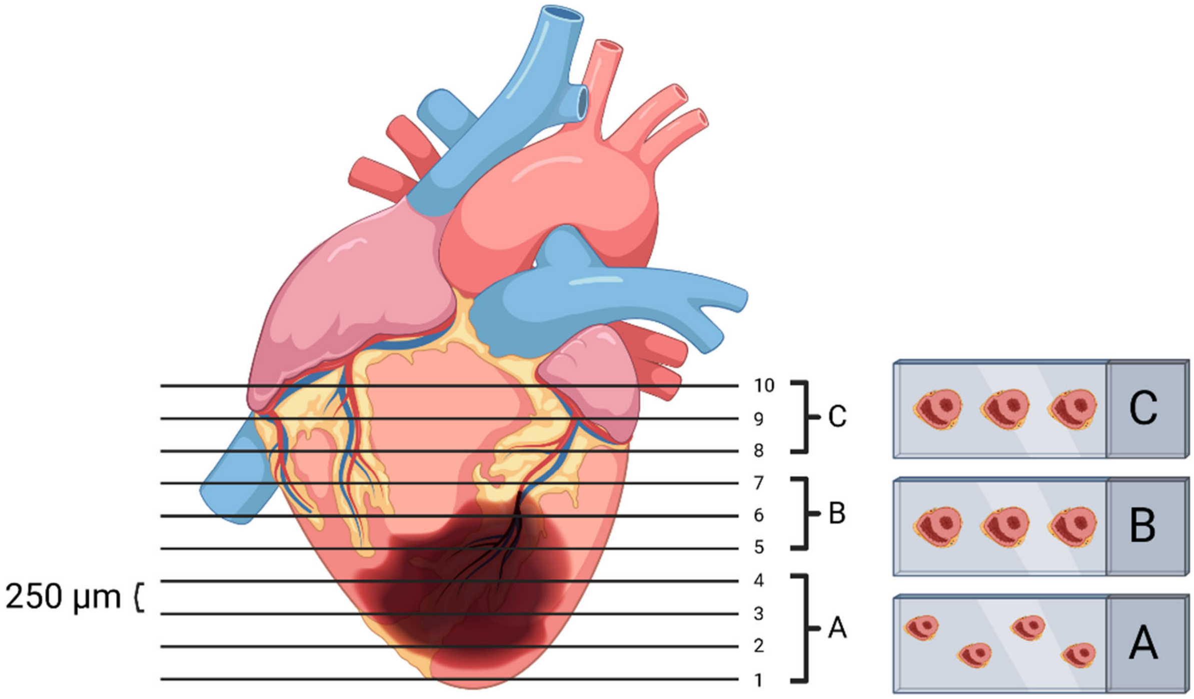

Supplementary Figure S1. Scheme for cryosectioning the heart. To visualize the infarct or scar area over the entire extension of the heart, sections are generated from 10 different levels. Starting at the apex of the heart, twenty separate slides with 5 µm-thick sections are prepared per level. A trimming step of 150 µm tissue is then performed before continuing with the next level, leading to a distance of 250 µm between each level (1-10). The sections of the different levels are distributed on slides (A: level 1-4, B: level 5-6, C: level 8-10). Created in BioRender. Elster, C. (2024) BioRender.com/q35v515

```
run("Duplicate...", "title=bild");
run("Gaussian Blur...", "sigma=8") //make tissue more homogenous//
run("Split Channels");
selectWindow("bild (blue)");
close();
selectWindow("bild (green)");
close();
selectWindow("bild (red)"); //red staining means only red channel has visible tissue//
setAutoThreshold("Triangle dark");
setThreshold(14, 255);
setOption("BlackBackground", false);
run("Convert to Mask"); //Makes image binary--> only way to be measured//
run("Measure");
```

Table S1. Semi-automated analysis of infarct size determination. ImageJ-macro for semi-automated measurement of phalloidin-stained tissue to determine infarct size at early time points (24 h) following experimental MI.

```
run("Duplicate...", "title=title");
run("Split Channels");
selectImage("title (green)");
close();
selectImage("title (red)");
setAutoThreshold("Default");
run("Threshold...");
setThreshold(18, 255);
setOption("BlackBackground", false);
run("Convert to Mask");
run("Measure");
close();
selectImage("title (blue)");
run("Convert to Mask");
run("Dilate");
run("Dilate");
run("Dilate");
run("Dilate");
run("Dilate");
run("Dilate");
run("Dilate");
run("Dilate");
run("Dilate");
run("Dilate");
run("Dilate");
run("Dilate");
run("Dilate");
run("Dilate");
run("Dilate");
run("Dilate");
run("Dilate");
run("Dilate");
run("Dilate");
run("Dilate");
run("Dilate");
run("Dilate");
run("Dilate");
run("Threshold...");
setThreshold(33, 255);
run("Convert to Mask");
run("Close");
run("Measure");
```

Table S2. Semi-automated analysis of area at risk vs. infarct area. ImageJ-macro for semi-automated analysis of Hoechst to phalloidin ratio. This was used to determine infarct area, area at risk and viable tissue 24h after experimental MI.

```
run("Fit Spline");
run("Duplicate...", "title=title");
run("Duplicate...", "title=title2");
selectWindow("title");
run("Split Channels");
selectWindow("title (blue)");
close();
selectWindow("title (green)");
close();
run("Gaussian Blur...", "sigma=8");
setAutoThreshold("Default dark");
run("Threshold...");
setThreshold(30, 255);
run("Convert to Mask");
run("Measure");
selectWindow("title2");
run("Split Channels");
selectWindow("title2 (blue)");
close();
selectWindow("title2 (red)");
close();
run("Gaussian Blur...", "sigma=8");
run("Threshold...");
setThreshold(35, 255);
run("Convert to Mask");
run("Measure");
```

Table S3. Semi-automated analysis of scare size determination. ImageJ-macro for automated measuring of scar size
